# PI3K/Akt/mTOR inhibitors in breast cancer

**DOI:** 10.7497/j.issn.2095-3941.2015.0089

**Published:** 2015-12

**Authors:** Joycelyn JX Lee, Kiley Loh, Yoon-Sim Yap

**Affiliations:** Department of Medical Oncology, National Cancer Center Singapore, Singapore 169610, Singapore

**Keywords:** Breast cancer, phosphoinositide 3 kinase (PI3K)/Akt/ mammalian target of rapamycin (mTOR), everolimus

## Abstract

Activation of the phosphoinositide 3 kinase (PI3K)/Akt/mammalian target of rapamycin (mTOR) pathway is common in breast cancer. There is preclinical data to support inhibition of the pathway, and phase I to III trials involving inhibitors of the pathway have been or are being conducted in solid tumors and breast cancer. Everolimus, an mTOR inhibitor, is currently approved for the treatment of hormone receptor (HR)-positive, human epidermal growth factor receptor 2 (HER2)-negative breast cancer. In this review, we summarise the efficacy and toxicity findings from the randomised clinical trials, with simplified guidelines on the management of potential adverse effects. Education of healthcare professionals and patients is critical for safety and compliance. While there is some clinical evidence of activity of mTOR inhibition in HR-positive and HER2-positive breast cancers, the benefits may be more pronounced in selected subsets rather than in the overall population. Further development of predictive biomarkers will be useful in the selection of patients who will benefit from inhibition of the PI3K/Akt/mTOR (PAM) pathway.

## Introduction

The phosphoinositide 3 kinase (PI3K)/Akt/mammalian target of rapamycin (mTOR) (PAM) pathway is frequently activated in breast cancer, with PIK3CA being the most commonly mutated gene of significance in estrogen receptor (ER) positive breast cancer^1^. Activation of the pathway has been associated with resistance to endocrine therapy, human epidermal growth factor receptor 2 (HER2)-directed therapy and cytotoxic therapy in breast cancer.

Several inhibitors of this pathway have been in preclinical development or in early phase clinical trials. Published data from randomised clinical trials is currently limited to mTOR inhibitors with everolimus, an mTOR inhibitor, being approved for treatment of hormone receptor (HR)-positive, HER2-negative advanced breast cancer. Other PAM pathway inhibitors which show promise include the PI3K inhibitors, Akt inhibitors, and the dual mTOR complex (mTORC) inhibitors.

Use of these inhibitors is associated with a unique spectrum of adverse effects which require special attention and management strategies.

In this article, we review the literature on the relevance of the PAM pathway in breast cancer, the advances in targeting this pathway including potential biomarkers and targets, and provide a practical approach to the toxicity management of mTOR inhibition.

## PAM pathway and its relevance in breast cancer

### The PAM pathway

**Figure 1:**
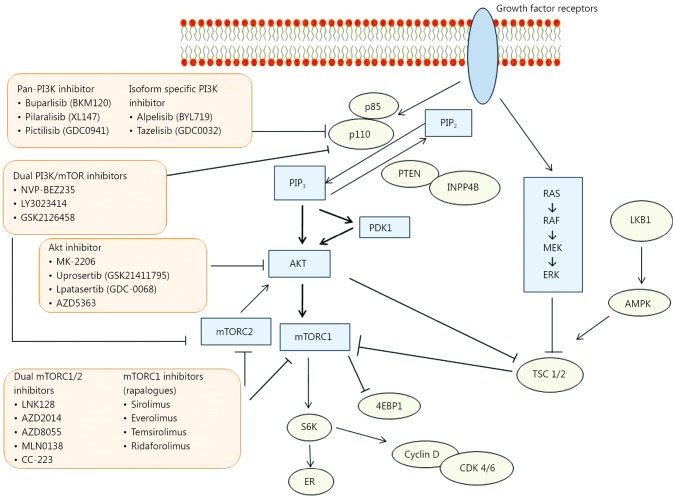
The PI3K/Akt/mTOR (PAM) pathway and inhibitors of the pathway tested in phase I-III clinical trials on solid tumors and/or breast cancer. PI3K, phosphoinositide 3 kinase; PTEN, phosphatase and tensin homologue deleted on chromosome ten; AKT, akt murine thymoma viral oncogene; mTORC, mammalian target of rapamycin complex; INPP4B, inositol polyphosphate 4-phosphatase II; 4EBP1, 4E-binding protein 1; TSC, tuberous sclerosis; RAS, rat sarcoma; RAF, rapidly accelerated fibrosarcoma; MEK, mitogen-activated protein kinase; ERK, extracellular signal-regulated kinase; LKB1, liver kinase B1; AMPK, AMP-activated protein kinase.

PAM is a major signalling pathway involved in cellular proliferation, survival, metabolism and motility. Studies suggest that the PI3K pathway is the most frequently altered pathway in human cancers, with *PIK3CA*^2^ and *PTEN*^3^ among the most frequently altered oncogenes and tumor suppressor genes respectively. Activation of the PAM pathway has been estimated to be in as frequent as 70% of breast cancers overall^4^ (**Table 1**, **Figure 1**).

**Table 1:** Common PAM pathway alterations in breast cancer

Gene	Type of alteration	Effect on signaling	Frequency (%)		
			HR+/luminal	HER2+	TNBC or basal-like
*PIK3CA*^5^	Activating mutation	Activation of PI3K signaling	28-47	23-33	8
*PTEN*	Loss-of-function mutation or reduced expression	Activation of PI3K signaling	29-44	22	67
*AKT1*	Activating mutation	Activation of AKT signaling	2.6-3.8	0	0
*AKT2*	Amplification	Activation of AKT signaling	2.8		
*PDK1*	Amplification or overexpression	Activation of AKT signaling	22	22	38
*INPP4B*^6^	Under-expression	Loss of regulation of AKT signaling	8	38	88
*LKB1*	Under-expression	Loss of regulation of AKT signaling	4.3-8.6^7^		
*ERBB2*	Amplification or overexpression	Activation of ErbB2 signaling (PI3K, MEK)	10	100	0
*IGF1R*	Receptor activation, *IGF1R* amplification	Activation of IGF-1R signaling (PI3K, MEK)	41-48	18-64	42
*FGFR1*	Amplification or activation mutation	Activation of FGFR signaling (PI3K, MEK)	8.6-11.6	5.4	5.6

Adapted from Miller *et al.*, *Breast Cancer Res* 2011^8^, *Agoulnik et al.*, *OncoTarget* 2011^6^; Hennessy *et al.*, *Cancer Res* 2009^5^; and Fenton *et al.*, *Appl Immunohistochem Mol Morphol* 2006^7^, with modifications. PAM, PI3K/Akt/mTOR; PIK3CA, phosphatidylinositol-kinase-3-catalytic-alpha; PTEN, phosphatase and tensin homologue deleted on chromosome ten; AKT, akt murine thymoma viral oncogene; PDK1, phosphoinositide-dependent kinase 1; INPP4B, inositol polyphosphate 4-phosphatase II; LKB, liver kinase B; ERBB2, erb-B2 avian erythroblastic leukemia viral oncogene homologue (also known as HER2, human epidermal growth factor receptor 2); IGF1R, insulin growth factor 1 receptor; FGFR, fibroblast growth factor receptor.

The PI3Ks, a family of lipid kinases, can be divided into three classes according to the structure, mode of regulation and lipid substrate specificity, of which the class I PI3K is related to cancer. Within class IA, the genes *PIK3CA*, *PIK3CB*, and *PI3KCD*, encode the homologous p110α, p110β, and p110δ isozymes respectively. Class IB consists of *PIK3CG*, which encodes p110γ ^9^. p110α and p110β are ubiquitously expressed while the expression of p110δ and p110γ is generally restricted to haematopoietic and immune cells. Class IA PI3Ks are heterodimeric proteins made up of a p110 catalytic subunit and a p85 regulatory subunit, and are involved in carcinogenesis. *PIK3CA* mutation occurs in approximately 35% of HR-positive breast cancers, in about 20%-25% of HER2-overexpressing breast cancers, and with a lower frequency in triple-negative breast cancers^10^ (**Table 1**).

PI3K is activated upstream by the binding of a growth factor or ligand to its cognate growth factor receptor tyrosine kinases (RTKs), which include members of the human epidermal growth factor receptor (HER) family, and the insulin and insulin-like growth factor 1 (IGF-1) receptor, among others^11^^,^^12^ (**Figure 1**). PI3K phosphorylates phosphatidylinositol 4,5-bisphosphate (PIP2) to phosphatidylinositol 3,4,5-triphosphate (PIP3), which in turn leads to phosphorylation of Akt, a serine/threonine kinase^13^. PIP3 acts as a docking site for AKT, which is the central mediator of the PI3K pathway and phosphoinositide-dependent kinase 1 (PDK1). Phosphorylation of AKT stimulates protein synthesis and cell growth by activating mTOR via effects on the intermediary tuberous sclerosis 1/2 complex (TSC1/2)^11^^,^^12^.

Phosphatase and tensin homologue deleted on chromosome ten (PTEN) is a tumor suppressor, which has inhibitory effects on the pathway by dephosphorylating PIP3 to PIP2. PIP3 levels are hence closely regulated by the opposing activities of PTEN and PI3K^14^. The role of inositol polyphosphate 4-phosphatase type II (INPP4B), another tumor suppressor, is increasingly recognised. INPP4B is also involved in dephosphorylation of PIP3 to PIP2^6^. Its loss has been reported as a marker of aggressive basal-like breast carcinomas^15^.

mTOR, a serine/threonine protein kinase, is a downstream effector of PI3K and Akt. It comprises two different complexes, mTOR complex 1 (mTORC1) and mTOR complex 2 (mTORC2), which are structurally similar but functionally different. mTORC1 is the target of rapamycin and rapamycin analogues, such as everolimus, and leads to cell anabolic growth by promoting mRNA translocation and protein synthesis^16^^,^^17^, and also has roles in glucose metabolism and lipid synthesis. Its downstream substrate S6 kinase 1 can phosphorylate the activation function domain 1 of the ER, which is responsible for ligand-independent receptor activation^18^^,^^19^. mTORC2 on the other hand, organises the cellular actin cytoskeleton and regulates AKT phosphorylation^20^. Rapalogues exert their effect mainly on mTORC1 and the incomplete inhibition can lead to feedback loops causing paradoxical activation of Akt and proliferative effects via other downstream targets.

Liver kinase B1 (LKB1) is a serine-threonine kinase upstream of AMP-activated protein kinase (AMPK), which in turn serves to negatively regulate mTOR signaling^21^ via TSC1 or 2 (**Figure 1**). LKB1, a tumor suppressor, is also known as serine/threonine kinase 11 (STK11), with germline mutations in *LKB1*/*SKT11* causing the Peutz-Jeghers tumor predisposition syndrome. Inactivation of the LKB1-AMPK pathway has been implicated in breast tumorigenesis^22^, and has also been associated with other cancers such as non-small cell lung cancer^23^^-^^25^ and hematologic malignancies^26^.

## Preclinical data

The PAM pathway has been implicated in endocrine resistance in preclinical breast cancer models^27^. Preclinical studies have shown that Akt can activate the ER pathway independent of estrogen availability and that the combination of mTOR inhibitors with endocrine therapy can overcome this resistance^28^^,^^29^.

In addition, the PAM pathway has also been implicated in trastuzumab resistance in HER2-overexpressing breast cancers^30^. As trastuzumab blocks the signaling pathway upstream from PI3K, a downstream aberration such as *PTEN* loss may override upstream inhibition. Preclinical studies indicate that inhibitors of the pathway can act synergistically with trastuzumab in resistant cells^31^^-^^34^.

In triple negative breast cancer (TNBC), preclinical studies including array comparative genomic hybridisation studies have shown that there is high frequency of loss of *PTEN* and *INPP4B*^35^^-^^37^, which correlates with Akt pathway activation. Everolimus has also been shown to sensitise basal-like breast cancer cells to DNA damaging agents, including cisplatin^38^^,^^39^, and to work synergistically with taxanes^40^.

## Clinical trials and predictive biomarkers

### mTOR inhibitors (Table 2)

**Table 2:** Summary of completed randomised trials of mTOR inhibitors in metastatic breast cancer

Study name	Comparison arms	Study description	Key findings	References
Hormone receptor Positive, HER2 negative				
HORIZON study	Temsirolimus + letrozole *vs.* placebo + letrozole	Phase III study, ABC, First-line (*n*=11,112)	PFS: 8.9 *vs.* 9.0 months (*P*=0.25); subgroup analysis: in <65 months, 9.0 *vs.* 5.6 months (*P*=0.003)	^41^
BOLERO-2 study	Everolimus + exemestane *vs.* placebo + exemestane	Phase III study, ABC, relapsed or progressed on previous NSAI (*n*=724)	Central PFS: 10.6 *vs.* 4.1 months (*P*<0.0001); local PFS: 6.9 *vs.* 2.8 months (*P*<0.0001); OS: 31.0 *vs.* 26.6 months (*P*=0.14)	^42^^,^^43^
TAMRAD study	Everolimus + tamoxifen *vs.* tamoxifen	Phase II randomised study; ABC; relapsed or progressed on previous AI (*n*=111)	CBR: 61% *vs.* 42% (*P*=0.045); TTP: 8.6 *vs.* 4.5 months (*P*=0.002)	^44^
HER2 positive				
BOLERO-3	Everolimus + vinorelbine + trastuzumab *vs.* placebo + vinorelibine + trastuzumab	Phase III study, ABC, previous treatment with taxane, resistance to trastuzumab (*n*=569)	PFS: 7.0 *vs.* 5.8 months (*P*=0.0067); subgroup analysis: PFS improved in HR- cancers but not in HR+ cancers	^45^
BOLERO-1	Everolimus + paclitaxel + trastuzumab *vs.* placebo + paclitaxel + trastuzumab	Phase III study, ABC, first-line (*n*=719)	PFS: 14.9 *vs.* 14.5 months (*P*=0.1167); however, in HR negative subpopulation: 20.3 *vs.* 13.1 months (*P*=0.0049)	^46^

ABC, advanced breast cancer; PFS, progression-free survival; NSAI, non-steroidal aromatase inhibitor; AI, aromatase inhibitor; CBR, clinical benefit rate; TTP, time to progression; HR, hormone receptor.

Rapamycin (sirolimus) was the first available mTOR inhibitor. It was initially developed and used as an immunosuppressant in transplant recipients. Temsirolimus was subsequently developed and is approved for the treatment of renal cell carcinoma. Everolimus is an oral mTOR inhibitor which has been approved for use in post-menopausal women with HR-positive breast cancer; it is also approved for used in other cancers including renal cell carcinoma, neuroendocrine tumors of the pancreas and subependymal giant cell astrocytomas. These agents are termed as “rapalogues” and work as allosteric inhibitors of mTORC1. However, in view that they inhibit only the mTORC1 complex, their use has been associated with negative feedback regulatory mechanisms and other mechanisms of resistance^47^, hence attenuating their efficacy in the single-agent setting.

#### HR positive, HER2 negative (HR+/HER2−) (HORIZON, BOLERO-2, TAMRAD)

The HORIZON trial was the first phase III randomised trial evaluating the use of an mTOR inhibitor in breast cancer. The trial compared the combination of temsirolimus plus letrozole to placebo plus letrozole, in the first-line setting in patients with HR+ advanced breast cancer. The study was terminated after an interim analysis showed that combination treatment did not improve progression-free survival (PFS) [median PFS, 8.9 *vs.* 9.0 months; hazard ratio (HR) =0.90; 95% CI, 0.76-1.07; *P*=0.25] and was associated with more grade 3 or 4 adverse events (37% *vs.* 24%)^41^.

The BOLERO-2 trial was another randomised phase III trial in advanced breast cancer evaluating an mTOR inhibitor and aromatase inhibitor (AI) combination. It randomised 724 postmenopausal women with HR-positive advanced breast cancer who had relapsed or progressed on nonsteroidal AI in a 2:1 ratio to exemestane plus everolimus (10 mg) *vs.* exemestane plus placebo. The addition of everolimus improved the PFS at central review to 10.6 months compared to 4.1 months with exemestane alone (*P*<0.0001), and the PFS at local review to 6.9 *vs.* 2.8 months (*P*<0.0001)^42^. These results led to the FDA approval in August 2012 of everolimus with exemestane in the treatment of postmenopausal women with HR+HER2− advanced breast cancer after failure of treatment with letrozole or anastrozole. The frequency of adverse events such as stomatitis, fatigue, non-infectious pneumonitis and hyperglycemia, and treatment discontinuations were higher in patients receiving the combination treatment. More recently, an update of the study reported overall survival (OS) of 31.0 months in the group receiving combination therapy compared to 26.6 months in the group receiving exemestane and placebo (*P*=0.14)^43^. The lack of significant improvement in OS, in spite of the increment in PFS, may be related to a number of issues. Firstly, the sample size was based on the primary endpoint of PFS and the trial was not statistically powered to detect an OS advantage of 4-6 months. There was also a small imbalance in the study groups with post-study salvage chemotherapy being more often used in the control arm. In addition, blockade of the mTORC1 complex by everolimus could have led to a negative intracellular feedback loop between the mTORC1 and IGF-1 signalling axis, leading to paradoxical activation of AKT. The higher rate of discontinuation of everolimus due to an adverse event, 29% compared to 5% in the control arm, may also have impacted treatment outcomes.

The TAMRAD study evaluated the addition of everolimus to tamoxifen in a phase II randomised study among HR+/HER2−, AI-resistant metastatic breast cancer patients. Addition of everolimus significantly improved the clinical benefit rate (CBR) as well as time to progression (TTP)^44^ and OS, although the study sample size was limited to 111 patients.

A subsequent analysis of the TAMRAD study demonstrated that patients with acquired or secondary resistance to endocrine therapy obtained more benefit from everolimus than patients with primary resistance. The differing results from the HORIZON study and the BOLERO-2 study suggest that patients with endocrine-resistant cancer may benefit more from the addition of an mTOR inhibitor, since the use of temsirolimus in the first-line setting in the former study failed to show benefit. Both BOLERO-2 and TAMRAD enrolled patients who had previously been treated with an AI while HORIZON was conducted with AI naïve patients and only 40% received adjuvant endocrine therapy. Given the potential toxicities with mTOR inhibitors, it is especially important to identify which patients would benefit from its use.

In addition to the above completed studies, there are other ongoing trials evaluating the use of mTOR inhibitors in other settings, such as in the adjuvant setting with endocrine therapy.

#### HER2 positive (HER2+) (BOLERO-3, BOLERO-1)

The BOLERO-3 trial was a phase III study which randomised patients previously treated with taxane and trastuzumab to everolimus 5 mg or placebo in combination with vinorelbine and trastuzumab^45^, testing the hypothesis that the addition of everolimus could overcome trastuzumab resistance. The addition of everolimus improved the PFS from 5.8 to 7.0 months (HR =0.78; *P*=0.0067). Subgroup analyses showed that PFS was significantly improved in patients with HR-negative cancers, but not with HR+ cancers, suggesting that ER+ breast cancers may be biologically different, and ER may act as an escape pathway when HER2 but not ER is inhibited. The role of everolimus in HER2+ breast cancer however remains unclear, especially with the approved indications for trastuzumab emtansine (TDM-1), lapatinib and pertuzumab. Of note, 28% of patients in the BOLERO-3 trial had previously received lapatinib.

The BOLERO-1 study was a phase III randomised study evaluating everolimus 10 mg in combination with paclitaxel and trastuzumab in HER2+ advanced breast cancer in the first-line setting, testing the potential for everolimus to circumvent trastuzumab resistance. The initial primary objective was investigator-assessed PFS in the full study population, with PFS in the subset of patients with HR-negative breast cancer added as a co-primary endpoint following the findings of the BOLERO-3 study. PFS in the full population was not statistically improved at 14.9 months in the group receiving everolimus compared to 14.5 months in the group receiving placebo (*P*=0.1167). In the HR-negative subpopulation, there was a 7.2 months prolongation in PFS with the addition of everolimus (20.3 *vs.* 13.1 months, *P*=0.0049)^46^, though the protocol-specified significance threshold (*P*=0.0044) was not crossed. Safety profile was consistent with results previously reported and included stomatitis, diarrhoea, neutropenia and anaemia. There was also a higher rate of adverse event-related on-treatment deaths with everolimus (3.6% *vs.* 0%) mainly related to respiratory problems and pneumonitis, again highlighting the need for proactive monitoring and early management of adverse events. The median everolimus relative dose intensity of 0.54 (range, 0.03-1.00) compared with 0.98 (range, 0.01-1.00) in the placebo group also reflects the difficulty in administering 10 mg of everolimus concurrently with weekly paclitaxel and trastuzumab.

#### Triple negative breast cancer (TNBC)

Following the encouraging preclinical data in TNBC, there have been a few randomised trials studying the use of everolimus in TNBC.

The first, a phase II neoadjuvant study including 50 TNBC patients, tested the addition of everolimus to weekly paclitaxel for 12 weeks, followed by fluorouracil, epirubicin and cyclophosphamide (FEC) every three weeks for four cycles. A higher clinical response rate was showed in the arm including everolimus (48% *vs.* 30%), but this did not reach statistical significance^48^. There was no significant improvement in pathological CR (pCR) in this small study.

The hypothesis of synergism between mTOR inhibitors and taxanes was also under investigation in a phase III study, where 403 patients with HER2- breast cancer showing no response after four cycles of neoadjuvant epirubicin and cyclophosphamide (with or without bevacizumab) were randomised to receive either paclitaxel alone, or paclitaxel plus everolimus. pCR was achieved in 3.6% of patients treated with paclitaxel and everolimus versus 5.6% in the control arm (*P*=0.476), making this a negative study^49^.

#### HER-2 negative metastatic breast cancer

In the metastatic setting, a randomised phase II trial evaluated the combination of paclitaxel and bevacizumab with or without everolimus in 112 women with untreated metastatic HER2-negative breast cancer. In a preliminary report, although response rates and median PFS were better with everolimus, the improvement in efficacy did not reach statistical significance, possibly attributed to the higher toxicities and lower dose intensity achieved in the everolimus arm^50^.

The negative results from the studies above highlight the lack of clinical efficacy in spite of promising preclinical activity. This discrepancy may be due to a number of issues, including toxicity affecting dose intensity, and activation of or crosstalk with other pathways *in vivo*, resulting in resistance to treatment.

### Biomarkers

Although the clinical efficacy of everolimus has been demonstrated in HR-positive and HER2-positive breast cancers, the benefits may be more pronounced in selected subsets rather than the overall population, as seen in the BOLERO and TAMRAD studies. To improve further drug development and to enrich the population that will benefit from PAM inhibitors, biomarkers of PAM activation, sensitivity and resistance to mTOR inhibitors need to be identified and validated.

Exon sequence and gene copy number variations were analysed in 182 cancer-related genes by next-generation sequencing (NGS) on archived tumor specimens from 227 patients in the BOLERO-2 study. A positive treatment effect in favour of everolimus was detected across the various genetic marker subgroups. *PIK3CA* mutation status was not predictive of increased benefit with addition of everolimus to exemestane. However, women whose tumors had two or more alterations in *PIK3CA* or *PTEN* or *FGFR1/2* or *CCND1* genes did not derive benefit in terms of PFS with everolimus. This suggests that concomitant alterations in more than one oncogenic pathway may attenuate everolimus efficacy^51^. One caveat to this biomarker subset analysis relates to the use of mainly archived primary tumor specimens rather than the latest metastatic specimens, which may not reflect the latest status with tumor evolution. In addition, there is a need to look beyond genomic alterations in the search for predictive biomarkers of everolimus efficacy.

Fifty-five primary tumor samples from the TAMRAD trial were evaluated for biomarkers, including tests for immunohistochemistry (IHC) on proteins that result in PAM pathway activation^52^. The subgroups most likely to have an improvement in TTP with tamoxifen/everolimus therapy, compared with tamoxifen alone, were patients with high p4EBP1 (a downstream effector of mTOR), low 4EBP1, low LKB1, low pAkt, and low PI3K. However, the small study sample size and multiple subgroup analyses mean that further validation in larger studies need to be done before implementation in the clinical setting for patient selection.

For HER2+ breast cancers, the preliminary results of a combined exploratory analysis on 377 samples from BOLERO-1 and BOLERO-3 trials were presented recently. Exons of 282 cancer related genes were analysed by NGS and PTEN levels were determined by IHC. Patients with hyperactive PI3K pathway (low PTEN or known *PIK3CA* or *AKT1* E17K mutation) benefitted from everolimus, whereas those without PI3K pathway activation did not (HR =0.76; 95% CI, 0.48-0.93; *P*=0.016)^53^.

The exploratory analyses as described have suggested that hyperactivation of the PI3K signalling pathway may possibly lead to preferential sensitivity to mTOR inhibitors, although validation studies are still needed

### PI3K/Akt inhibitors

A different strategy of targeting the PAM pathway involves the inhibition of upstream targets such as PI3K and Akt (**Figure 1**). Many of these compounds have only reached the stage of early phase trials. The isoform selectivity and other pharmacologic properties may vary from compound to compound. While there are dual inhibitors which inhibit both PI3K and mTOR, further development may be limited by issues such as increased toxicity.

Currently, the only data from randomised trials is from the phase II FERGI trial, which evaluated the role of adding pictilisib (GDC0941), a class I PI3K inhibitor, to fulvestrant. A total of 168 women with ER+ advanced breast cancer who had progressed on prior AI use were randomised to fulvestrant (500 mg monthly) with pictilisib (340 mg daily) or fulvestrant with placebo. The preliminary results were presented at San Antonio Breast Cancer Symposium 2014^54^; the addition of pictilisib to fulvestrant was associated with a non-statistically significant PFS increase from 5.1 to 6.6 months (HR =0.74; *P*=0.096). *PIK3CA* mutation status did not predict the benefit of addition of pictilisib to fulvestrant either, but this was based on archived tumor specimens, which may not reflect the latest mutation status and underscores the fact that *PI3K* genotype may not be the most reliable biomarker of response.

BKM120, or buparlisib is another PI3K inhibitor which is more advanced in clinical development. Buparlisib is an oral selective inhibitor of pan-class I PI3K, which equally inhibits class IA PI3Ks, but has no activity against class III PI3Ks or Mtor^55^. The BELLE-2 study (Clinicaltrials.gov no: NCT01610284) is a phase III trial which randomised 1,148 postmenopausal women with HR+/HER2- advanced breast cancer after progression on AI to fulvestrant and buparlisib or fulvestrant and placebo; preliminary results may soon be available. This study also evaluates the role of PI3K pathway activation with both *PIK3CA* mutation status and PTEN loss on IHC on archival tumor samples, as well as mutation status based on circulating tumorDNA. Another trial, the BELLE-3 study (Clinicaltrials.gov no: NCT01633060), looks at the same treatment combination in patients who have progressed after an AI and mTOR inhibitor^56^.

More recent ongoing trials involve the alpha-selective PI3K inhibitors such as BYL719 and GDC0032, which may provide more specific inhibition of PIK3CA than the pan-PI3K inhibitors, allowing for maintenance of efficacy while limiting toxicity from off-target effects^57^.

With the current trends of personalised precision medicine, there is increasing emphasis on biomarker development and selection of patients with PAM pathway activation for the newer trials.

### Overcoming resistance and novel combinations

The PI3K pathway involves a complex network of interactions with many parallel cascades, so its inhibition releases negative feedback resulting in activation of compensatory signalling pathways^58^, including PTEN loss^59^. In addition, given the heterogeneous genomic architecture of breast cancers^1^, there are often multiple drivers in different pathways, such that PI3K-AKT may not be the dominant regulator of mTOR in some cells^60^.

To overcome this, combination therapy regimens have been or are being tested in both the preclinical and clinical settings. These include combination with RTK inhibitors such as those directed against EGFR, HER2^61^^,^^62^ or HER3; and combination with MEK inhibitors, to overcome the parallel induction of the MAPK pathway, though this strategy may improve efficacy at the expense of increased toxicity^63^. Activating feedback loops involving insulin growth factor 1-receptor (IGF-1R)/PI3K that occur during mTOR inhibition, resulting in Akt and mitogen-activated protein kinase (MAPK) activation, have been implicated in secondary resistance^64^. The combination of temsirolimus and an anti-IGF-1 receptor antibody, cixutumumab, has been tested in a phase I trial with 26 metastatic breast cancer patients^65^. Stable disease was the best response observed, and data from an ongoing phase II study is awaited (Clinicaltrials.gov: NC802077933). Combination vertical blockade of both PI3K and mTOR is also being evaluated in the phase II trial of BYL719 together with everolimus and exemestane (Clinicaltrials.gov no: NCT02077933).

Rapalogues are postulated to have limited efficacy as single-agents in view of their incomplete mTOR inhibition, hence newer pathway inhibitors are being developed, such as INK128, a dual mTORC1/mTORC2 inhibitor, which was studied in a phase I trial with acceptable toxicities^66^. AZD2014, another dual mTORC1/mTORC2 inhibitor, showed promise of clinical efficacy and tolerability in a phase I study^67^, and is now being studied in a four-arm phase II study in two different dosing schedules together with fulvestrant alone or fulvestrant and everolimus (Clinicaltrials.gov no: NCT02216786).

More recently, preclinical data suggests that CDK 4/6 inhibition may act synergistically with PI3K inhibition to reduce cell viability and overcome intrinsic and adaptive resistance mechanisms^68^. Current phase I/II studies are being conducted using a combination of CDK4/6 inhibitor and PI3K inhibitors, such as the combination of LEE011 with fulvestrant and BYL719 or BKM120 in postmenopausal women with advanced HR+ breast cancer (Clinicaltrials.gov no: NCT02088684), and another study is looking at the combination of LEE011, BYL719 and letrozole (Clinicaltrials.gov no: NCT01872260).

## Toxicities

PAM inhibitors are associated with certain class-effect toxicities such as hyperglycaemia and rash. Most of the monitoring and management guidelines are currently limited to everolimus or mTOR inhibitors, as trials on other PAM inhibitors are still ongoing. While most of the adverse effects may be only mild to moderate in severity, education of healthcare professionals and patients is crucial in ensuring patient safety and compliance. An important example is the early recognition of pneumonitis, as this is a potentially life-threatening complication when severe. A proposed algorithm for the monitoring of potential side effects is shown in **Table 3**. Details on the management of adverse effects associated with everolimus may also be obtained at http://www.global.afinitor.com and https://www.pharma.us.novartis.com/product/pi/pdf/afinitor.pdf.

**Table 3:** Recommended monitoring guidelines for a patient on everolimus

Item	Detailed guidelines
Pre-treatment screening	Screen baseline full blood count, renal panel, liver panel, fasting glucose, lipid panel; screen baseline virologies for hepatitis B and other opportunistic infections as clinically indicated;
	Screen baseline O_2_ saturation and lung imaging;
	No dose adjustment is needed for renal impairment, but is required for hepatic impairment
Advice to patients at start of treatment	Once daily dosing at same time every day, consistently either with or without food;
	Tablets should be swallowed whole with water, should not be chewed or crushed;
	Advise patients on potential adverse events including pneumonitis (cough, breathlessness), infections (fever, localising symptoms), hypersensitivity (breathlessness, flushing, rash, swelling), oral ulceration, and hyperglycemia (and reinforce need for monitoring if patient is already a known diabetic);
	Advise patients regarding potential drug interactions and to inform any physician they see that they are on this drug;
	Drugs to avoid include moderate to strong inhibitors of cyp3a4 (e.g., ketoconazole, clarithromycin etc.) and moderate to strong inducers of CYP3A4 (e.g., carbamazepine, phenytoin, St John’s wort etc.) as well as moderate to strong inhibitors or inducers of P-glycoprotein (PgP);
	Advise patients on need for contraception and to avoid breast-feeding
Monitoring during treatment	Review patient every 1-2 weeks for first month of initiation;
	Periodic monitoring of full blood count, renal panel, liver panel, fasting glucose; suggest to repeat 2 weeks and 4 weeks after initiation of treatment and periodically (every 4-6 weeks) thereafter;
	Lipid panel may be checked periodically e.g., every 6-8 weeks initially

The following toxicities are most commonly reported within everolimus^69^.

### Cutaneous and mucosal effects

Stomatitis was the most common adverse event reported in both the TAMRAD and the BOLERO-2 trials. It is clinically distinct from conventional chemotherapy-associated mucositis, being characterised by aphthous ulcerations and grey-white pseudomembranous changes. In addition to good oral hygiene, management may also include the use of corticosteroid mouth rinses or everolimus dose modifications (**Table 4**). With appropriate management, everolimus may be continued in some patients who experience stomatitis^70^.

**Table 4:** Management of common side effects from mTOR inhibitors and required dose adjustments (partly adapted from www.global.afinitor.com)

Grading	Description	Suggested management
Management of stomatitis		
Grade 1	Minimal symptoms, normal diet; erythema of mucosa	Alcohol-free mouthwash
Grade 2	Symptomatic but can tolerate modified diet; patchy ulcerations or pseudomembranes	Topical treatments including local anaesthetic mouthwash, with or without corticosteroids; interrupt treatment until resolution to grade 1 or less, then reinitiate at 10 mg (first occurrence), 5 mg (second occurrence)
Grade 3	Symptomatic; unable to tolerate orally; confluent ulcerations or pseudomembranes	Topical treatments including local anaesthetic mouthwash, with or without steroids; interrupt treatment until resolution to grade 1 or less, then reinitiate at 5 mg (first occurrence); consider discontinuation if there is grade 3 recurrence
Grade 4	Symptomatic, life-threatening tissue necrosis, significant spontaneous bleeding	Discontinue treatment; supportive treatment as above
Management of rash		
Grade 1	Macular or papular eruption or erythema; asymptomatic	Topical treatments including low potency corticosteroids and moisturisers; symptomatic treatment e.g., antihistamines
Grade 2	Symptomatic eruption or erythema (e.g., pruritus), localised desquamation or other lesions covering <50% body surface area (BSA)	Topical treatments including low potency corticosteroids and moisturisers; symptomatic treatment e.g., antihistamines; interrupt treatment until resolution to grade 1 or less, then reinitiate at 10 mg (first occurrence), 5 mg (second occurrence)
Grade 3	Severe, generalised erythroderma or eruption/desquamation covering >50% BSA	As above for management of rash + systemic steroids ± antibiotics; interrupt treatment until resolution to grade 1 or less, then reinitiate at 5 mg (first occurrence); consider discontinuation if there is grade 3 recurrence
Grade 4	Generalised exfoliative, ulcerative or bullous dermatitis	As above for management of rash; discontinue treatment
Management of non-infectious pneumonitis		
Grade 1	Asymptomatic; radiographic findings only	Observation including use of imaging; dose adjustment not required
Grade 2	Symptomatic; ADLs not impaired	Rule out infection; consider treatment with steroids; consult pulmonologist; interrupt treatment until resolution to grade 1 or less, then reinitiate at 5 mg; discontinue if there is no resolution within 4 weeks
Grade 3	Symptomatic; ADLs impaired; oxygen required	Rule out infection; treatment with steroids; consult pulmonologist; interrupt treatment until resolution to grade 1 or less, then reinitiate at 5 mg; discontinue if there is no resolution within 4 weeks
Grade 4	ADLs severely impaired; mechanical ventilation required; life-threatening	Rule out infection; treatment with corticosteroids; consult pulmonologist; discontinue treatment
Management of metabolic effects		
Grade 1	FG > ULN-8.9 mmol/L; HC > ULN-7.75 mmol/L; HTG > ULN-2.5× ULN	No dose adjustment; monitor and treat hyperglycemia/dyslipidemia as appropriate
Grade 2	FG >8.9-13.9 mmol/L; HC >7.75-10.34 mmol/L; HTG >2.5-5.0× ULN	No dose adjustment; monitor and treat hyperglycemia/dyslipidemia as appropriate
Grade 3	FG >13.9-27.8 mmol/L HC >10.34-12.92 mmol/L HTG >5.0-10× ULN	Interrupt treatment until resolution to grade 1 or less, then reinitiate at 5 mg; monitor and treat hyperglycemia/dyslipidemia as appropriate
Grade 4	FG >27.8 mmol/L; HC >12.92 mmol/L; HTG >10× ULN	Discontinue treatment; monitor and treat hyperglycemia/dyslipidemia as appropriate

FG, fasting glucose; ULN, upper limit normal; HC, hypercholesterolemia; HTG, hypertriglyceridemia.

Everolimus is also associated with an acneiform rash that may require topical corticosteroids, with or without topical antibiotics, and antihistamines. Severe cases may require systemic corticosteroids and antibiotics, as well as dose interruption, reduction or discontinuation.

### Metabolic effects

mTOR inhibitors may cause hyperglycemia and hyperlipidemia, with elevations in both low density lipoprotein (LDL) cholesterol and triglycerides. Everolimus is contraindicated in patients with uncontrolled diabetes and requires optimisation of glycemic control prior to initiation^71^. Recommended management of the metabolic effects of the PAM pathway inhibitors is summarised in **Table 4**.

### Non-infectious pneumonitis

Non-infectious pneumonitis is an inflammatory reaction that is usually gradual in onset, and associated with radiologic findings of ground-glass opacities and focal consolidation. Management depends on symptom severity, and may require referral to a pulmonologist, corticosteroids and drug discontinuation if severe (see **Table 4**). Empirical treatment for infection is often indicated, as it is a common differential diagnosis and can co-exist with drug-related pneumonitis. Early recognition and treatment of this potentially life-threatening complication is crucial.

### Immunosuppression

Patients on everolimus can be predisposed to infections, including bacterial, fungal and viral infections, as well as reactivation of hepatitis B virus. Baseline screening for hepatitis B, and other infections such as hepatitis C, HIV and tuberculosis should be considered before drug initiation for patients at risk of reactivation with prior exposure or certain risk factors.

### Constitutional

Everolimus is also associated with increased incidence of all-grade fatigue^72^, asthenia and anorexia^42^. The management is largely supportive, with psychosocial support, physical therapy and nutritional supplementation. Dose reduction may be indicated in severe cases where quality of life is adversely affected.

## Conclusion

The PAM pathway is frequently activated in breast cancer, and inhibitors targeting this pathway are currently available or being tested in clinical trials. Data of clinical efficacy is mainly in the setting of HR+/HER2− breast cancer at this present moment, with everolimus approved for use in combination with exemestane after progression on non-steroidal AIs. In HER2+ disease, benefit appears to be limited to the HR- subset, although PAM pathway activation status also appears to be predictive of everolimus efficacy.

Monitoring and timely management of adverse effects are critical to minimise toxicities and optimise efficacy from this class of therapeutics. Future directions include optimising efficacy with novel combinations to overcome resistance mechanisms, as well as further development of predictive biomarkers for better selection of patients who will benefit from PAM inhibitors.
